# Maternal nitrate supplementation improves offspring cardiometabolic outcomes in obese pregnancies

**DOI:** 10.1080/07853890.2025.2521440

**Published:** 2025-07-07

**Authors:** Kristi M. Crowe-White, Katelyn E. Senkus, Janie C. DiNatale, Arlin B. Blood, Taiming Liu, Meijuan Zhang, Khondoker Adeba Ferdous, Rebecca Bloch, Han-A Park

**Affiliations:** ^a^ Department of Human Nutrition, The University of Alabama, Tuscaloosa, AL, USA; ^b^ USDA/ARS Children’s Nutrition Research Center, Department of Pediatrics, Baylor College of Medicine, Houston, TX, USA; ^c^ Loma Linda University School of Medicine, Loma Linda, CA, USA

**Keywords:** Nitrate supplementation, cardiometabolic outcomes, pregnancy, overweight, obesity, offspring, preclinical study

## Abstract

**Objective:**

Obesity during pregnancy is associated with excess adiposity and metabolic abnormalities in offspring due to fetal programming. Although the impact of dietary nitrate on cardiometabolic health has been established, it is unknown if maternal dietary nitrate intake would exhibit similar benefits to offspring. This study assessed cardiometabolic outcomes in offspring of Sprague-Dawley rats fed a high fat diet (HFD) with or without inorganic nitrate.

**Methods:**

Eight-week-old female rats (*n* = 11) were randomized to 10% normal fat diet (NFD) or 45% HFD. Upon confirmation of obesity, some HFD rats (*n* = 4) were transitioned to HFD with 40 mg inorganic nitrate (HFDN). NFD (*n* = 4), HFD (*n* = 3), and HFDN (*n* = 4) maintained their respective diets, yet all weanling pups were transitioned to NFD at post-natal day 21 (P21). Body composition was assessed by bioimpedance spectroscopy along with assessment of body weight (BW), and abdominal circumference as well as brown adipose tissue (BAT) and white adipose tissue (WAT) mass. Serum nitrate, cardiometabolic biomarkers (glucose, insulin, total cholesterol, HDL-cholesterol, and triglycerides, along with serum redox status), blood pressure, and protein levels of F_1_Fo ATP synthase c-subunit in BAT were assessed.

**Results:**

Prior to mating, HFD and HFDN females exhibited significantly greater BW and fat mass (*p* = 0.01) compared to the NFD group. Serum nitrate was significantly higher in HFDN dams, yet there were no significant differences in maternal cardiometabolic biomarkers between groups. At P21, BW was significantly lower in pups reared by NFD and HFDN dams compared to those reared by HFD dams (*p* = 0.044, *p* = 0.027, respectively). Abdominal circumference and glucose were significantly lower in HFDN pups compared to NFD (*p* = 0.004, *p* = 0.003, respectively) and HFD pups (*p* = 0.011, *p* = 0.006, respectively). At P65, abdominal circumference was lower in HFDN pups compared to NFD and HFD pups, albeit non-significantly. HFDN pups also exhibited significantly lower triglycerides compared to HFD pups (*p* = 0.004). Additionally, pups born to HFD-fed dams exhibited significantly decreased protein levels of F_1_Fo ATP synthase c-subunit in pups at both P21 and P65, yet nitrate supplementation significantly reversed the effects of the HFD at P21 (*p* = 0.046) and P65 (*p* = 0.0056).

**Conclusions:**

Results suggest periconceptional and prenatal nitrate supplementation may beneficially impact cardiometabolic outcomes of offspring born from an overweight or obese pregnancies including body weight, abdominal circumference, glucose dynamics, and lipid profiles. Further, results suggest that maternal intake of dietary nitrate impacts cellular responses during fetal development, thus regulating energy metabolism of offspring. .

## Introduction

Excess adiposity during gestation may induce perturbations in offspring that impact adipose tissue accrual and function as well as long-term cardiometabolic health [1,2]. These insults on the fetus are further exacerbated by the composition of the maternal diet. For example, the Developmental Origins of Health and Disease Theory suggests that epigenetic influences *in utero* such as the prenatal diet may influence fetal development during critical windows [2]. Thus, excess adiposity coupled with a poor diet during gestation may induce cellular and developmental alterations in offspring through a process known as metabolic programming [3,4]. Such changes detrimentally influence adipose tissue differentiation resulting in increased deposition of white adipose tissue (WAT) in offspring. In addition to serving as the primary storage site for triglycerides, WAT is an active endocrine organ that promotes systemic oxidative stress and inflammation, thus, fostering an environment conducive to the onset of cardiometabolic disease [5]. Taken collectively, this perfect storm in the health of women of reproductive age equates to an increased risk of obesity and cardiometabolic disease across the lifespan of both mother and child [6]. Thus, obesity in pregnancy represents a critical public health concern warranting further investigation and intervention.

Albeit understudied, deficiency of nitric oxide (NO) – a physiological vasodilator - is among the mechanisms underpinning the metabolic perturbations of obesity including impaired endothelial function, hypertension, diabetes, and hypercholesterolemia among others [7]. Conversely, adequate NO production is associated with decreased weight gain and visceral fat accumulation as well as improvements in cardiometabolic health [8]. In addition to the endogenous production of NO from L-arginine, dietary inorganic nitrate is an exogenous substrate for NO production *via* the nitrate-nitrite-NO pathway [9]. Acknowledging the beneficial effects of diets rich in nitrate such as the Dietary Approaches to Stopping Hypertension (DASH) diet, it is unknown if adherence to a maternal diet rich in nitrate during the periconceptional and prenatal period can influence the nutritional programming of offspring adipose tissue (AT) development and functionality as well as attenuate cardiometabolic disease risk**.** With approximately 50% of women entering pregnancy while overweight or obese, the critical need for research on nutritional programming has been recently prioritized by the National Institutes of Health. According to the 2020–2030 Strategic Plan for Nutrition Research, Strategic Goal 3 Objective 3.1 calls for examining the role of periconceptional and prenatal nutrition in development and disease outcomes.

It should be noted that dietary nitrates have been mischaracterized in previous years as detrimental to human health due to the potential of nitrate salts in cured and processed meats to form *N*-nitrosamines under elevated temperatures; however, recent studies considering only plant food sources of dietary nitrate have consistently reported cardiovascular health benefits to the extent that dietary nitrate is being considered as a conditionally essential nutrient for vascular health [10]. Further, it has been established that risks associated with other sources of nitrate (namely processed foods and drinking water) are not evident with plant-based dietary sources.

To date, the influence of dietary nitrate on measures of adiposity and cardiometabolic health has been evaluated in several adult rodent models [11–13]; however, given the epigenetic potential for dietary modifications to prevent or mitigate harmful metabolic programming [1], it is critical to expand the window of investigation to periods of fetal development. As this study is the first of its kind, the aim of this study was to investigate periconceptional and prenatal intake of dietary nitrate on body composition, AT accrual and function as well as cardiometabolic health of offspring born to obese Sprague-Dawley rats consuming a high fat diet with or without inorganic nitrate. As pregnancy is a highly protected time of research participation, studies of this kind represent the first step in investigating dietary interventions during pregnancy, thus leading to future translational nutrition research.

## Methods

### Experimental animals and study design

In this preclinical study, female Sprague-Dawley rats (*n* = 11, eight weeks old) were purchased from Charles River Laboratories (Wilmington, MA, USA). Animals were individually housed in a climate-controlled environment (23 °C ± 1 °C, 12h light-12h dark cycle) with free access to food and water. Following a one-week acclimation period, animals were randomized to a purified diet with 10% kcal from fat (normal fat diet, NFD) or a purified diet with 45% kcal from fat (high fat diet, HFD) to induce obesity (Research Diets, New Brunswick, NJ, USA). Obesity was confirmed by significant differences in body weight and fat mass according to diet-induced obesity curves using the ImpediVET^TM^ bioimpedance spectroscopy device (ImpediMed Inc., Carlsbad, CA, USA) [14]. Upon obesity confirmation, HFD rats (*n* = 4) were transitioned to an HFD supplemented with 40 mg inorganic nitrate (HFDN) for approximately four weeks before mating and during pregnancy to represent supplementation during the periconceptional and prenatal periods (Universal Preserv-A-Chem Inc., Mebane, NC, USA). This dose was determined based on dietary nitrate provided by adherence to the DASH diet [15]. Nitrate consumed from adherence to this diet has been reported to range from 174 to 1222 mg nitrate depending on the foods selected; thus, 600 mg dietary nitrate was selected and principles of allometric scaling were employed [16].

Dough treats were used to deliver the intervention, yet all animals were trained to consume plain 1 g of dough treat daily (Bio-Serv, Flemington, NJ, USA). Each gram of dough provided 3.83 calories comprised of 46.5% carbohydrate, 21.2% protein, 12.4% fat, and 4.8% fiber. For female rats consuming NFD and HFD without inorganic nitrate, 1 g of plain dough treat was provided daily. For HFDN female rats, inorganic nitrate powder was added to the center of each 1 g of dough treat daily. Dough treats (1 g) were prepared and fed to each animal daily with confirmation of full consumption visually obtained. NFD (*n* = 4), HFD (*n* = 3), and HFDN (*n* = 4) rats maintained their respective diets and dough treat allocation throughout gestation, lactation, and weaning.

**Figure 1.**
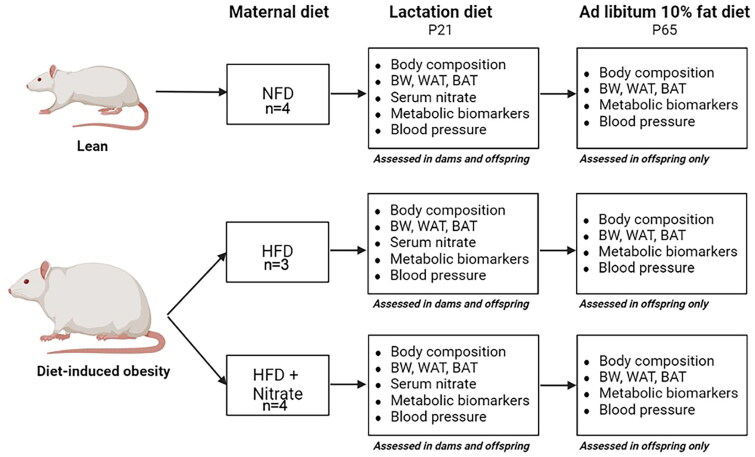
Experimental study procedures. Animals were randomized to a purified diet with 10% kcal from fat (normal fat diet, NFD) or a purified diet with 45% kcal from fat (high fat diet, HFD) to induce obesity. Following confirmation of obesity, HFD rats (*n* = 4) were transitioned to a HFD supplemented with 40 mg inorganic nitrate. The others continued NFD or HFD without inorganic nitrate supplementation through lactation. Beginning at P21, all remaining pups were transitioned to NFD without inorganic nitrate supplementation. Each box in the figure depicts which outcome measures were assessed at each euthanasia time point. P21 and P65, postnatal day 21 and 65, respectively; BW: body weight; WAT: white adipose tissue; BAT: brown adipose tissue.

Animals were monitored daily throughout the study, and body weights and dietary intake were assessed bi-weekly. Specifically, dietary intake was assessed by weighing the food provided and consumed for each female rat two times per week. All procedures followed the National Institutes of Health’s Guide for the Care and Use of Laboratory Animals and were approved by the Institutional Animal Care and Use Committee of the University of Alabama (IACUC# 20-10-4066) (Tuscaloosa, AL, USA). Further, all reporting herein complies with the ARRIVE guidelines for reporting of *in vivo* experiments with animals. The day of parturition was defined as postnatal day 0 (P0). At P21, weanling pups from the respective groups were transitioned to NFD only. Pups were euthanized at P21 and P65 representing the weanling and young adult phases of the lifecycle [17]. Dams were euthanized following weaning confirmation at P21. Outcome measures were assessed for dams and pups with the intervals of testing depicted in Figure 1.

### Body composition

For both dams and pups, body composition was assessed using an ImpediVET^TM^ bioimpedance spectroscopy device (ImpediMed Inc., Carlsbad, CA, USA) as previously described [18]. Briefly, anesthetized animals were positioned on their abdomen with fore and hind limbs extended distally on a non-conductive surface. Four needle electrodes were inserted sub-dermally along the rat’s dorsal midline according to manufacturer guidelines. Five consecutive measures were conducted, and the average total body water, fat-free mass, fat mass, and body mass index were calculated. Additionally, abdominal circumference was measured at the largest section of the rat’s abdomen using a plastic measuring tape [19].

### Blood pressure

Rats were anesthetized using an intraperitoneal injection of ketamine (80 mg/kg body weight) and xylazine (5 mg/kg body weight) (Patterson Veterinary, Loveland, CO, USA). A 20-minute resting period was implemented for each rat to ensure sedation stabilization before beginning blood pressure assessment. Tail-cuff blood pressure was measured non-invasively using the BP-2000-R2 Blood Pressure Analysis System (Visitech Systems, Inc. Apex, NC, USA) [20].

### Blood and tissue collection

Following blood pressure and body composition assessment, rats were euthanized *via* carbon dioxide inhalation. Blood collected from the vena cava was centrifuged, and the obtained serum was stored at −80 °C until analysis. WAT and interscapular brown adipose tissue (BAT) were harvested, weighed, and snap-frozen in liquid nitrogen for storage at −80 °C until analysis. Additionally, the ratio of BAT mass to WAT mass was also calculated [21].

### Serum nitrate analysis

Serum nitrate concentrations were determined using enzymatic reduction of nitrate to nitrite by incubation with nitrate reductase enzyme (Roche, Indianapolis, IN, USA) at 37 °C for 45 min followed by a previously described triiodide ozone-based chemiluminescence assay [22].

### Cardiometabolic measures

Serum glucose, total cholesterol, HDL-cholesterol, and triglycerides were measured using an automated SIRRUS analyzer (Stanbio Laboratory, Boerne, TX, USA). Minimum sensitivities and intra-assay CV% for glucose, total cholesterol, HDL-cholesterol, and triglycerides were 2 mg/dl and 1.28%, 5 mg/dl and 1.33%, 5 mg/dl and 6.1%, and 2.0 mg/dl and 1.11%, respectively. Insulin was measured using a Millipore Sensitive Rat Insulin RIA kit (Billerica, MA, USA) with a 0.09 ng/ml minimum sensitivity and 6.30% intra-assay CV.

Antioxidant and lipid peroxide extraction of BAT was conducted according to a previously validated method [23]. BAT was then deproteinated using methanol/acetonitrile/acetone (1:1:1, v/v/v) and the oxygen radical absorbance capacity assay on a FLUOstar Optima plate reader (BMG Labtech, Cary, NC, USA) was used to measure hydrophilic and lipophilic antioxidant capacity [24,25]. The compound 2,2-azobis(2—amidino-propane) dihydrochloride was used as the peroxyl radical generator and Trolox, a water-soluble analogue of vitamin E, served as the reference antioxidant standard. Antioxidant capacity results are expressed as uM Trolox equivalents.

Malondialdehyde, a product of lipid peroxidation and a biomarker of oxidative stress, was quantified in BAT using the thiobarbituric acid reactive substances assay as previously described [26]. Oxidative stress results are expressed as mM malondialdehyde.

### Immunoblots

Samples were lysed in RIPA buffer (Cell signaling Technology, Danvers, MA), and protein concentration was determined using BCA protein reagents (Thermo Scientific, Rockford, IL). Samples (50–70 µg of protein/lane) were separated on a 4%–12% SDS-polyacrylamide gel (Bio-Rad, Hercules, CA) and probed with anti-ATP5G1/G2/G3 (1:500, Abcam, Cambridge, UK), anti-UCP1 (1:100, Santa Cruz Biotechnology, Dallas, TX), and anti-beta actin (1:1000, Sigma-Aldrich, St. Louis, MO). Scanned images were analyzed using ImageJ software (National Institutes of Health, Bethesda, MD).

### Statistical analysis

The normality of data was confirmed *via* skewness and kurtosis values. Mean differences between groups were assessed using one-way ANOVA followed by Bonferroni post-hoc testing. Significance was defined as *p* < 0.05. Data are presented as mean ± standard deviation with post-hoc values reported narratively. All analyses were performed using SPSS Statistics version 29 (SPSS, Inc., Chicago, IL, USA).

## Results

### Maternal outcomes

Female rats receiving NFD weighed significantly less than rats fed either HFD or HFDN prior to mating (*p* = 0.01) (Table 1). Maternal fat mass was significantly different between groups with NFD dams having less fat mass than both HFD and HFDN dams both prior to mating and at necropsy (pre-mating: *p* = 0.010; necropsy: NFD vs. HFD *p* < 0.001 and NFD vs. HFDN *p* = 0.004). In addition to the noted differences in body composition, maternal rats fed NFD had significantly less WAT than dams fed HFD or HFDN (NFD vs. HFD *p* = 0.034, NFD vs. HFDN *p* = 0.029). NFD dams also had less BAT than rats fed HFD (NFD vs. HFD *p* = 0.041). Maternal HFDN had significantly more serum nitrate than NFD and HFD (NFD 1.94 ± 0.7 μM, HFD 1.38 ± 0.58 μM, HFDN 7.98 ± 2.32 μM; NFD vs. HFDN *p* = 0.003, HFD vs. HFDN *p* = 0.006).

**Table 1.** Maternal body composition.

	NFD (*n* = 4)	HFD (*n* = 3)	HFDN (*n* = 4)	*p* value
**Prior to Mating**				
Body Weight (g) (mean ± SD)	283.3 ± 34.9	406.7 ± 59.2	385.3 ± 13.0	0.010*^§^
Body Mass Index (g/cm^2^) (mean ± SD)	11.1 ± 1.6	15.3 ± 0.3	15.3 ± 1.3	0.165
Fat Mass (%)(mean ± SD)	45.7 ± 3.7	51.5 ± 3.1	46.9 ± 5.4	0.010*^§^
Fat-Free Mass (%)(mean ± SD)	54.7 ± 3.1	48.5 ± 3.1	53.1 ± 5.4	0.605
**Necropsy (Post-Natal Day 21)**				
Body Weight (g) (mean ± SD)	296.8 ± 35.6	444.9 ± 56.4	437.6 ± 23.1	<0.001*^§^
Body Mass Index (g/cm^2^) (mean ± SD)	12.5 ± 1.6	15.4 ± 2.0	15.0 ± 2.6	0.900
Fat Mass (%) (mean ± SD)	27.8 ± 13.6	50.6 ± 7.6	45.3 ± 1.6	<0.001*^§^
Fat-Free Mass (%) (mean ± SD)	72.2 ± 13.6	49.4 ± 7.6	54.8 ± 1.6	0.034*^§^
WAT (g) (mean ± SD)	16.0 ± 10.0	56.7 ± 16.3	52.3 ± 7.7	0.001*^§^
BAT (g) (mean ± SD)	0.45 ± 0.2	1.0 ± 0.4	0.8 ± 0.2	0.041*

NFD: normal fat diet; HFD: high fat diet; HFDN: high fat diet with nitrate supplementation; WAT: white adipose tissue; BAT: brown adipose tissue.

*p* < 0.05 denotes significance.

*significant differences exist between NFD and HFD.

^§^ significant differences exist between NFD and HFDN.

Despite differences in body composition, there were no significant differences between groups for any of the cardiometabolic outcome measures among dams. Additionally, there were no significant differences between groups for litter size (number of pups per litter) or average pup weight at week one; however, HFD dams had significantly more pup deaths within one week after birth than observed in NFD litters (*p* = 0.001) and HFDN litters (*p* = 0.01) (Table 2).

**Table 2.** Litter size, weight, and deaths of offspring born to NFD, HFD, and HFDN dams.

Variables	NFD Dams (*n* = 4)	HFD Dams (*n* = 3)	HFDN Dams (*n* = 4)	*p* value*
Litter Size (*n*)	7.3 ± 3.8	6.7 ± 3.1	7.3 ± 4.0	0.973
Average Pup Weight** (g)	19.4 ± 3.7	13.5 ± 0.6	17.1 ± 2.1	0.068
Number of Deaths After Birth (*n*)	1.0 ± 0.8	5.7 ± 1.2	2.3 ± 1.3	0.001*

NFD: normal fat diet; HFD: high fat diet; HFDN: high fat diet with nitrate supplementation.

* *p* < 0.05 denotes significance with exact post-hoc values reflected in the narrative.

** Average pup weight measured one week after birth.

### Offspring outcomes by necropsy time point

#### Post-natal day 21

**Figure 2.**
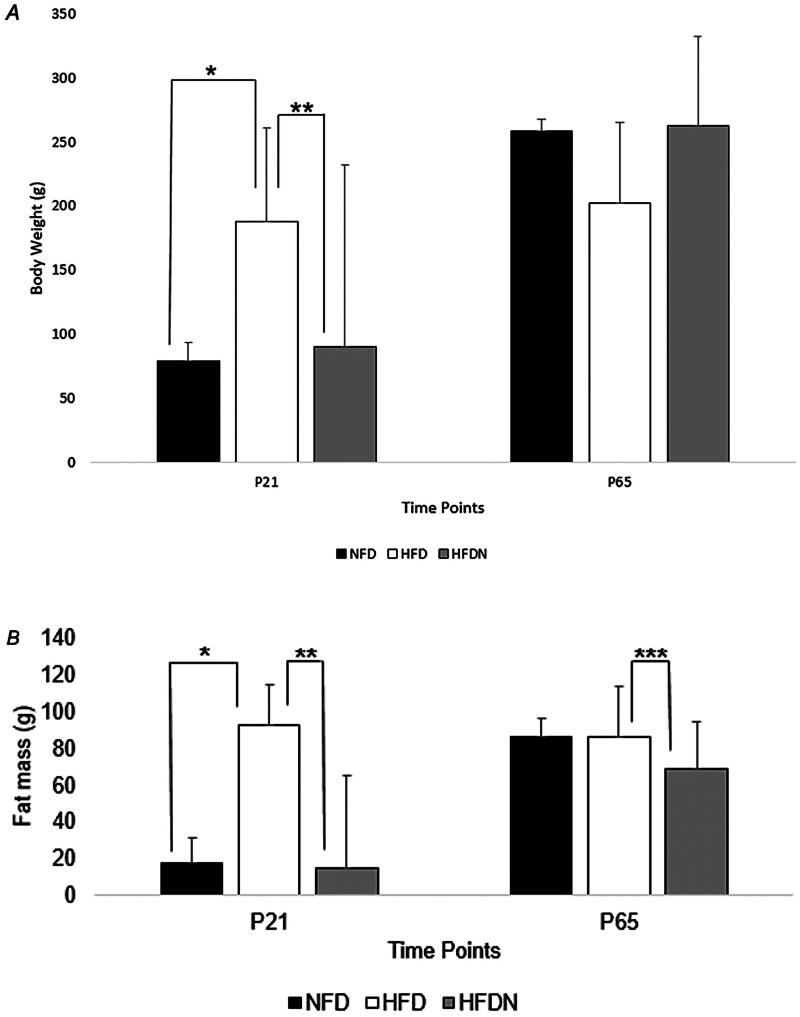
(A) Offspring body composition. (A) Offspring body weight at each necropsy time point. *p = 0.044; **p = 0.027. NFD: normal fat diet; HFD: high fat diet; HFDN: high fat diet with nitrate supplementation; P: postnatal day. P21: NFD *n* = 6; HFD *n* = 12; HFDN *n* = 5. P65: NFD *n* = 5; HFD *n* = 3; HFDN *n* = 11. (B) Offspring fat mass as assessed by bioimpedance spectroscopy at each necropsy time. *p = 0.049; **p = 0.042; ***p = 0.007. NFD: normal fat diet; HFD: high fat diet; HFDN: high fat diet with nitrate supplementation; P: postnatal day. P21: NFD *n* = 5; HFD *n* = 8; HFDN *n* = 3 P65: NFD *n* = 10; HFD *n* = 3; HFDN *n* = 11.

At P21, body weight was significantly different between groups such that NFD pups weighed significantly less than HFD pups (*p* = 0.044) and HFD pups weighed significantly more than HFDN pups (*p* = 0.027) (Figure 2A). Fat mass assessed by bioimpendance spectroscopy was significantly lower in NFD offspring compared to HFD offspring (*p* = 0.049) and significantly lower in HFDN offspring compared to HFD offspring (*p* = 0.042) (Figure 2B). NFD and HFD pups also had significantly greater abdominal circumference compared to HFDN pups at this time point (NFD 12.02 ± 0.66 cm, HFD 13.43 ± 2.64 cm, HFDN 9.92 ± 0.7 cm; NFD vs. HFDN *p* = 0.004, HFD vs. HFDN *p* = 0.011).

**Figure 3.**
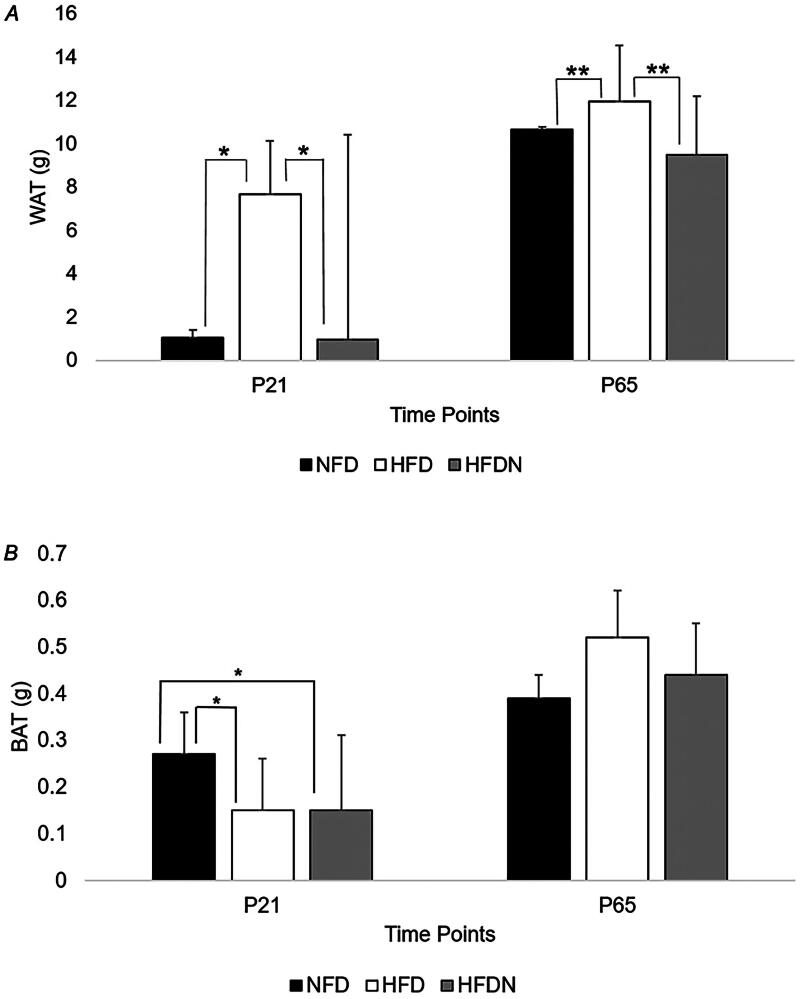
(A) Offspring adipose tissue measurements. *p < 0.001; **p = 0.01. NFD, normal fat diet; HFD, high fat diet; HFDN, high fat diet with nitrate supplementation; WAT, white adipose tissue. P21: NFD *n* = 6; HFD *n* = 12; HFDN *n* = 5. P65: NFD *n* = 11; HFD *n* = 3; HFDN *n* = 11. (B) Offspring brown adipose tissue. *p < 0.001. NFD: normal fat diet; HFD: high fat diet; HFDN: high fat diet with nitrate supplementation; BAT: brown adipose tissue. P21: NFD *n* = 6; HFD *n* = 12; HFDN *n* = 5. P65: NFD *n* = 12; HFD *n* = 3; HFDN *n* = 11.

WAT was significantly lower in pups reared by NFD and HFDN dams compared to pups reared from HFD dams (NFD vs. HFD and HFD vs. HFDN *p* < 0.001 both) (Figure 3A). Additionally, significant differences were observed between groups for BAT such that NFD pups had significantly more BAT than HFD and HFDN pups (*p* < 0.001 both) (Figure 3B). When normalizing BAT to body weight (BAT:BW), significant differences persisted between groups where NFD had a lower BAT:BW ratio than HFDN (*p* = 0.019), and HFD had lower BAT:BW than HFDN pups (*p* = 0.004). Additionally, BAT:WAT was significantly higher in NFD compared to HFD pups (*p* = 0.007) and significantly higher in NFD compared to HFDN pups (*p* = 0.004).

Systolic blood pressure was not significantly different between groups at P21, yet diastolic blood pressure was significantly different (*p* = 0.025). Upon post-hoc analysis, no significant differences were observed between groups. Nevertheless, HFDN pups exhibited a 23% and 25% reduction in diastolic blood pressure compared to NFD and HFD pups, respectively. With regard to glucose as a metabolic biomarker, both NFD and HFD pups had significantly greater serum glucose levels compared to HFDN pups at P21 (NFD 654.33 ± 4.04 mg/dL, HFD 646.75 ± 21.58 mg/dL, HFDN 457.67 ± 98.08 mg/dL; NFD vs. HFDN *p* = 0.003, HFD vs. HFDN *p* = 0.006). Significant differences between groups for other cardiometabolic measures or serum nitrate were not observed at post-natal day 21.

#### Post-natal day 65

At P65, body weight was not significantly different between groups (Figure 2A). However, pups reared from HFDN dams had significantly less fat mass compared to pups reared from HFD dams (*p* = 0.007) (Figure 2B). Additionally, HFDN pups had smaller abdominal circumference measurements than both NFD and HFD pups albeit non-significant (*p* = 0.05).

NFD and HFDN pups had significantly less WAT than HFD pups at P65 (NFD vs. HFD and HFD vs. HFDN *p* = 0.01, both) (Figure 3A). Pups from HFD dams also had more BAT than pups reared from NFD and HFDN dams, although these results were not statistically significant (*p* = 0.05, Figure 3B). When normalizing BAT to body weight, no significant differences existed at P65; however, there were significant differences in BAT:WAT with pups reared from HFDN dams having less BAT:WAT compared to pups reared from HFD dams (NFD 0.10 ± 0.11, HFD 0.08 ± 0.08, HFDN 0.05 ± 0.01, *p* = 0.010).

With regard to cardiometabolic outcomes, significant differences existed in initial analyses between groups for HDL-cholesterol (*p* = 0.049); however, upon post-hoc analysis, differences were not significant (NFD 51.00 ± 5.79 mg/dL, HFD 59.00 ± 1.73 mg/dL, HFDN 55.75 ± 3.86 mg/dL). Additionally, significant differences in serum total cholesterol were observed such that NFD pups had less total cholesterol than HFD pups (NFD 71.80 ± 20.84 mg/dL, HFD 82.33 ± 16.12 mg/dL, HFDN 75.25 ± 18.01 mg/dL; *p* = 0.038) and HFDN pups had significantly lower triglycerides than HFD pups (NFD 186.40 ± 48.07 mg/dL, HFD 234.00 ± 50.69 mg/dL, HFDN 160.25 ± 48.99 mg/dL; *p* = 0.004). For all other cardiometabolic outcomes assessed, no significant differences were observed at post-natal day 65.

**Figure 4.**
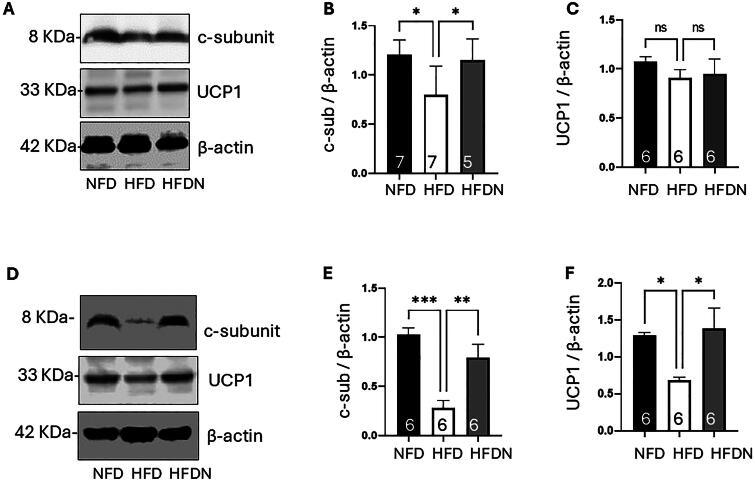
Offspring brown adipose tissue protein assessment. Offspring BAT protein as assessed by immunoblotting at P21 (A–C) and P65 (D–F). (A) Immunoblot data using BAT isolated at P21 show that treatment with nitrate prevents the loss of the F_1_Fo ATP synthase c-subunit (B), but UCP-1 was not changed (C). (D) Immunoblot data from P65 showed that treatment with nitrate prevent the loss of both the F_1_Fo ATP synthase c-subunit (E) and UCP-1 (F). *p < 0.05, **p < 0.01, and ***p < 0.001. NFD: normal fat diet; HFD: high fat diet; HFDN: high fat diet with nitrate supplementation; BAT: brown adipose tissue

To understand the role of nitrate in energy metabolism, selected mitochondrial proteins in BAT were quantified (Figure 4). Although the levels of uncoupled protein 1 (UCP-1) were unchanged (NFD 1.07 ± 0.13, HFD 0.91 ± 0.21, HFDN 0.83 ± 0.25), HFD significantly decreased the protein levels of the F_1_Fo ATP synthase c-subunit in P21 pups. In addition, nitrate treatment reversed the effects of HFD (NFD 1.20 ± 0.15, HFD 0.80 ± 0.30, HFDN 1.15 ± 0.22; NFD vs. HFDN *p* = 0.117, HFD vs. HFDN *p* = 0.046), suggesting that nitrate plays a role in preventing the loss of mitochondrial uncoupling channel. Notably at P65, the groups showed greater effects of nitrate on both proteins. Treatment with nitrate prevented the reduction of F_1_Fo ATP synthase c-subunit (NFD 1.03 ± 0.17, HFD 0.28 ± 0.18, HFDN 0.79 ± 0.33; NFD vs. HFDN *p* = 0.0002, HFD vs. HFDN *p* = 0.0056) and UCP-1 (NFD 1.29 ± 0.11, HFD 0.68 ± 0.11, HFDN 1.39 ± 0.68; NFD vs. HFDN *p* = 0.048, HFD vs. HFDN *p* = 0.021) under HFD.

## Discussion

Results of this study suggest that nitrate supplementation in the periconceptional and prenatal window may lead to significant improvements in offspring body composition, adipose tissue function, and cardiometabolic measures compared to rats born from an overweight or obese pregnancy without supplementation of nitrate. With results observed at P21 and P65, these timepoints are reflective of the acute and chronic imprinting effects of maternal dietary intake on offspring.

While several meta-analyses have been published highlighting the inverse association of dietary nitrate with cardiovascular disease and type 2 diabetes in adults [27–29], this study is the first of its kind to investigate maternal supplementation of dietary nitrate on cardiometabolic effects in offspring. Further, this is the first study assessing periconceptual and prenatal dietary nitrate supplementation. Acknowledging that infants born to obese, overweight, and diabetic mothers (even when normal weight) have increased adiposity and are at increased risk of later metabolic disease [3], results of this study suggest that an intervention to bolster intake of dietary nitrate may address, to some extent, such adverse cardiometabolic programming of offspring.

In the current study, decreases in body weight, abdominal circumference, and WAT accrual were observed in HFDN pups. Similarly, Carlstrom et al. reported significant reductions in visceral fat accumulation in adult mice as a result of nitrate intake while Bakhtiarzadeh et al. reported significant reductions in body weight, adiposity indices, BMI, and abdominal circumference in nitrate-treated rats in response to oral supplementation [12,13]. Potential mechanisms underpinning these results include the nitrate-induced browning effect of WAT with substantial increases in oxygen consumption and fatty acid β-oxidation in adipocytes [11]. Additional mechanisms by which nitrate disrupts adiposity accrual and prevents associated metabolic complications include regulation of adipocyte mitochondrial respiration and oxidative phosphorylation efficiency [30,31].

In this study, we suggest a nitrate-mediated mitochondrial mechanism that contributes to the regulation of cellular energy metabolism. The F_1_Fo ATP synthase c-subunit has been previously shown to form an uncoupling channel that alters cellular energy metabolism whereas approaches to deplete the c-subunit have been revealed to increase energy production [32,33]. We found that maternal HFD decreased the c-subunit and UCP-1 protein in P65 pups, which suggests that maternal HFD impacts the cellular responses of offspring, potentially favoring energy production. Treatment with nitrate reversed the effect of HFD, indicating the energy expenditure regulation effects of nitrate.

Although the effects of decreased diastolic blood pressure did not remain significant upon post-hoc analysis, nitrate supplementation has been shown to decrease systolic and diastolic blood pressure and subsequent CVD risk in a recent systematic review and meta-analysis [34]; such reductions may translate into achievable improvements in the blood pressure-related burden of cardiovascular disease [35].

Additionally, metabolic improvements were observed in offspring from HFDN dams such that serum glucose was significantly lower compared to offspring from NFD and HFD dams at P21; further at P65, triglycerides in offspring from HFDN moms were significantly lower than offspring from HFD dams. As all offspring were transitioned upon weaning to a 10% fat diet (NFD) without nitrate supplementation, results reflect maternal imprinting and metabolic fetal programming. Nevertheless, although nitrate was not directly consumed by the offspring in this study, Li et al. reported similar decreases in blood glucose and dyslipidemia upon the addition of nitrate to diets of HFD + fructose fed mice [36] while Zand et al. reported reductions in triglycerides upon supplementation with nitrate and nitrite for four weeks [37]. As such, results support the role of nitrate in cardiometabolic health both indirectly and directly.

Collectively, mechanisms underpinning nitrate functionality include NO generation by way of the nitrate-nitrite-nitric oxide pathway [38]. As NO regulates blood flow and oxygen perfusion to cells and tissues, the generation of NO from dietary nitrate represents a primary therapeutic target for restoring imbalances in blood pressure, inflammation, oxidative stress, and immune dysfunction as well as diminished NO production related to obesity. Thus, interventions to improve the intake of dietary nitrate in the periconceptional and prenatal window of fetal development may mitigate harmful metabolic programming of maternal adiposity and high fat dietary patterns. Further research is needed to understand the mechanistic underpinnings of the beneficial impacts of maternal dietary nitrate intake on offspring and on the timing of nitrate supplementation before, during, and/or after pregnancy (pre- or post-natal supplementation).

Strengths of this study include the controlled dosing of nitrate and the attainability of the human equivalency dose of 600 mg nitrate/d through adherence to the DASH diet or consumption of beetroot juice [39]. As this is the first study of its kind to evaluate the effects of maternal nitrate supplementation on the cardiometabolic health of offspring, this research underscores the need for food-based guidelines to capitalize on the role of dietary nitrate in NO homeostasis for metabolic and vascular health across the lifespan [10]. Despite the strengths of this study, it is not without inherent limitations, most notably, difference in litter sizes resulting in different number of offspring outcomes assessed at P21 and P65.

## Conclusions

Dietary nitrate supplementation may beneficially impact cardiometabolic outcomes of offspring from an overweight or obese pregnancy including body weight, WAT accrual, abdominal circumference, glucose dynamics, and lipid profiles; further, results corroborate previous findings indicating that the gestational period is optimal for dietary interventions targeting maternal and offspring cardiometabolic outcomes. Taken collectively, increasing nitrate consumption through adherence to the DASH diet or selecting foods rich in nitrate could be a practical and efficacious approach to improving the metabolic health of offspring from infancy to later in life. Lastly, as obesity in pregnancy represents a critical public health concern, the detrimental clinical implications for both mothers and offspring may be mitigated by existing dietary patterns rich in nitrate such as the DASH diet.

## Data Availability

Data that support the findings of this study are available from the corresponding author upon reasonable request and consideration.
